# Intensity-modulated radiotherapy for cushing’s disease: single-center experience in 70 patients

**DOI:** 10.3389/fendo.2023.1241669

**Published:** 2023-09-26

**Authors:** Xin Lian, Zhuoran Xu, Shuai Sun, Weiping Wang, Huijuan Zhu, Lin Lu, Xiaorong Hou, Fuquan Zhang

**Affiliations:** ^1^ Department of Radiation Oncology, State Key Laboratory of Complex Severe and Rare Diseases, Peking Union Medical College Hospital, Chinese Academy of Medical Science and Peking Union Medical College, Beijing, China; ^2^ Department of Endocrinology, State Key Laboratory of Complex Severe and Rare Diseases, Peking Union Medical College Hospital, Chinese Academy of Medical Science and Peking Union Medical College, Beijing, China

**Keywords:** cushing’s disease, intensity-modulated radiotherapy, radiotherapy, pituitary adenoma, ACTH

## Abstract

**Context:**

Intensity-modulated radiotherapy (IMRT) is a modern precision radiotherapy technique for the treatment of the pituitary adenoma.

**Objective:**

Aim to investigate the efficacy and toxicity of IMRT in treating Cushing’s Disease (CD).

**Methods:**

70 of 115 patients with CD treated with IMRT at our institute from April 2012 to August 2021 were included in the study. The radiation doses were usually 45-50 Gy in 25 fractions. After IMRT, endocrine evaluations were performed every 6 months and magnetic resonance imaging (MRI) annually. Endocrine remission was defined as suppression of 1 mg dexamethasone test (DST) or normal 24-hour urinary free cortisol level (24hUFC). The outcome of endocrine remission, endocrine recurrence, tumor control and complications were retrieved from medical record.

**Results:**

At a median follow-up time of 36.8 months, the endocrine remission rate at 1, 2, 3 and 5 years were 28.5%, 50.2%, 62.5% and 74.0%, respectively. The median time to remission was 24 months (95%CI: 14.0-34.0). Endocrine recurrence was found in 5 patients (13.5%) till the last follow-up. The recurrence-free rate at 1, 2, 3 and 5 years after endocrine remission was 98.2%, 93.9%, 88.7% and 88.7%, respectively. The tumor control rate was 98%. The overall incidence of new onset hypopituitarism was 22.9%, with hypothyroidism serving as the most common individual axis deficiency. Univariate analysis indicated that only higher Ki-67 index (P=0.044) was significant favorable factors for endocrine remission.

**Conclusion:**

IMRT was a highly effective second-line therapy with low side effect profile for CD patients. Endocrine remission, tumor control and recurrence rates were comparable to previous reports on FRT and SRS.

## Introduction

Cushing’s disease (CD) is characterized by hypersecretion of adrenocorticotropic hormone (ACTH) from pituitary adenoma. As the state of hypercortisolemia considerably increases morbidity and mortality, normalizing cortisol levels is regarded as the major treatment goal in patients with CD (1). Transsphenoidal selective adenomectomy (TSS) is now established as the first-line treatment of CD. Despite the satisfactory remission rate that can be achieved with TSS (ranging from 59-97%), delayed recurrences have also been reported in up to 50% of patients (2).

The Endocrine Society guidelines suggest a shared decision-making approach in patients who underwent a noncurative surgery or for whom surgery was not possible (3). Second-line therapeutic options include repeat transsphenoidal surgery, medical therapy, radiotherapy and bilateral adrenalectomy. Radiotherapy (RT) is generally used in patients who have failed TSS or have recurrent CD, as well as in progressively growing or invasive corticotroph tumors (3, 4).

Both stereotactic radiosurgery(SRS)and fractionated radiotherapy (FRT) have been used in the treatment of CD. Conventional radiotherapy as one of the technique for FRT has been used with a long experience, but its benefits were hindered by high risk of toxicity, mainly attributed to the harm to healthy surrounding structures (4). Previous studies on conventional RT in treating CD showed high efficacy (tumor control rate of 92-100% and hormonal control rate of 46-89%), but RT-induced hypopituitarism (30-58%) and recurrence (16-21%) were also commonly reported (1, 4–7). Modern precise radiotherapy, especially intensity-modulated radiotherapy (IMRT), can spare the surrounding normal structure better by a more conformal and precise dose distribution (8). However, a large cohort study on long-term efficacy and toxicity of IMRT for CD is still lacking. Therefore, in the current study, we aim to analyze the efficacy and toxicity of intensity-modulated radiotherapy (IMRT) in treating CD. We also investigated the predictors of endocrine remission in aid of further management.

## Methods

### Patient

We collected 115 cases of Cushing’s disease treated at our center from April 2012 to August 2021. Patients were excluded under the following conditions: (1) follow-up time less than 3 months, (2) lacking evaluation of serum cortisol (F), adrenocorticotropic hormone (ACTH) or 24-hour urinary free cortisol (24hUFC) before or after RT, (3) underwent uni or bilateral adrenalectomy, (4) having received RT at other institutes before admitted to our center. At last, a total of 70 cases were included in this study.

### Radiotherapy parameters

RT was administrated by a linear accelerator (6 MV X-ray). Intensity-modulated radiation therapy was applied for all patients. Including fix-filde IMRT (FF-IMRT), volumetric modulated arc therapy (VMAT) or Tomotherapy. We immobilized the patient with an individualized thermoplastic head mask and then conducted a computed tomography (CT) simulation scan at 2- to 3-mm intervals. The target volume and organs at risks (OARs) were delined with a contrast enhanced T1-weighted image (T1WI) magnetic resonance imaging (MRI) fusing with planning CT. The gross tumor volume (GTV) was defined with the lesion visible on MRI or CT. The clinical target volume (CTV) included microscopic disease, especially when the tumor invaded cavernous sinus and surrounding bones. The planning target volume (PTV) was defined as CTV plus a margin of 2- to 3-mm in three dimensions. The prescription dose was defined at 100% isodoseline to cover at least 95% PTV. The maximum dose was limited to less than 54 Gy for the brain stem and optic pathway structures. Radiotherapy was performed once a day and five fractions a week during five to six weeks. The total dose was 45-60 Gy, delivered in 25-30 fractions, with most patients (78.6%) receiving 45-50 Gy in 25 fractions. The fractionated dose was 1.8-2.0 Gy.

### Data collection and clinical evaluation

Baseline characteristics were collected at the last outpatient visit before RT, including demographic characteristics, biochemical data, tumor characteristics and details of previous treatments. After RT, endocrine evaluations were performed every 6 months. Endocrine remission was considered when 1 mg dexamethasone suppression test (DST)<1.8 mg/dl. If 1mg DST results were lacking, then 24hUFC within the normal range was used as a remission criterion. Patients who regained elevated hormone levels after achieving remission were considered to have endocrine recurrence. For patients receiving medications that could interfere with the metabolism of cortisol, hormonal evaluation was performed at least 3 months after the cessation of the therapy.

Tumor size was measured on magnetic resonance imaging (MRI) before RT and annually after the completion of RT. Any reduction in or stabilization of tumor size was considered as tumor control. Tumor recurrence was defined as an increase of 2 millimeters in 2 dimensions comparing to MRI before RT, or from invisible tumor to a visible tumor on MRI (9).

Anterior pituitary function was assessed before RT and every 6 months during the follow-up after RT. RT-induced hypopituitarism was defined as the development of new onset hormone deficiency after RT. The diagnostic criteria for growth hormone deficiency (GHD), central hypothyroidism and hypogonadotropic hypogonadism (HH) refer to previous literature (10–12). Panhypopituitarism referred to three or more anterior pituitary hormone deficiencies (13).

### Statistical analysis

Statistical analysis was performed with SPSS version 25.0. Longitudinal analysis was performed with Kaplan-Meier method. For time-dependent variable, Log rank test was used for univariate analysis and Cox regression for multivariate analysis. The cut-off of F, ACTH and 24hUFC were defined as their median value. All variants in the univariate analysis were included in the model of multivariate analysis. P value < 0.05 was considered statistically significant. Plot was created with GraphPad Prism version 9.4.

## Results

### Patient characteristics

Of 70 cases included in the study, the median age was 32 years (range, 11-66 years). 60 (85.7%) were female and 10 (14.3%) were male (F:M= 6:1). The median follow-up time was 36.8 months (range, 3.0-111.0 months). 68 patients received RT as a second-line treatment because of incomplete tumor resection, failure to achieve complete endocrine remission or recurrence postoperative, and 2 were treated with RT alone because of contraindication of surgery. The frequency of surgical treatment was 1 for 42 patients, 2 for 21 and more than 3 for 5. A total of 8 patients received medical treatment before RT. 5 of them used pasireotide, 2 used ketoconazole and 1 used mifepristone. The median ACTH level was 58.7 pg/ml (range 14.9-265 pg/ml), F, 26.2μg/dl (range 11.8-72.6 μg/dl) and 24hUFC, 355.7 μg/24hr (range 53.5-3065 μg/24hr) before RT. Tumor size evaluation was performed in all 70 patients before RT. Among them, 36 patients showed no visible residual tumor identified on MRI and only 5 patients showed tumor size more than 1 cm. Hypopituitarism was found in 31 patients (38.8%) before RT. HH was the most common (21 patients, 26.3%), followed by central hypothyroidism (13 patients, 16.3%) and GHD (9 patients, 11.3%). Panhypopituitarism was found in 4 patients (5.0%). (**Table 1**).

**Table 1:** Patient characteristics.

	No. of patients (%)
Sex	
Male	10 (14.3)
Female	60 (85.7)
Tumor on MRI	
No visible mass	36 (51.4)
Visible mass	34 (48.6)
Surgery	
0	2 (2.9)
1	42 (60)
2	21 (30)
≥3	5 (7.1)
Medication before RT	
No	62 (88.6)
Yes	8 (11.4)
Hypopituitarism before RT	
Yes	31 (44.3)
No	39 (55.7)
Ki-67 index	
< 3%	32 (45.7)
≥ 3%	20 (28.6)
Unknown	18 (25.7)

### Endocrine remission

**Figure 1:**
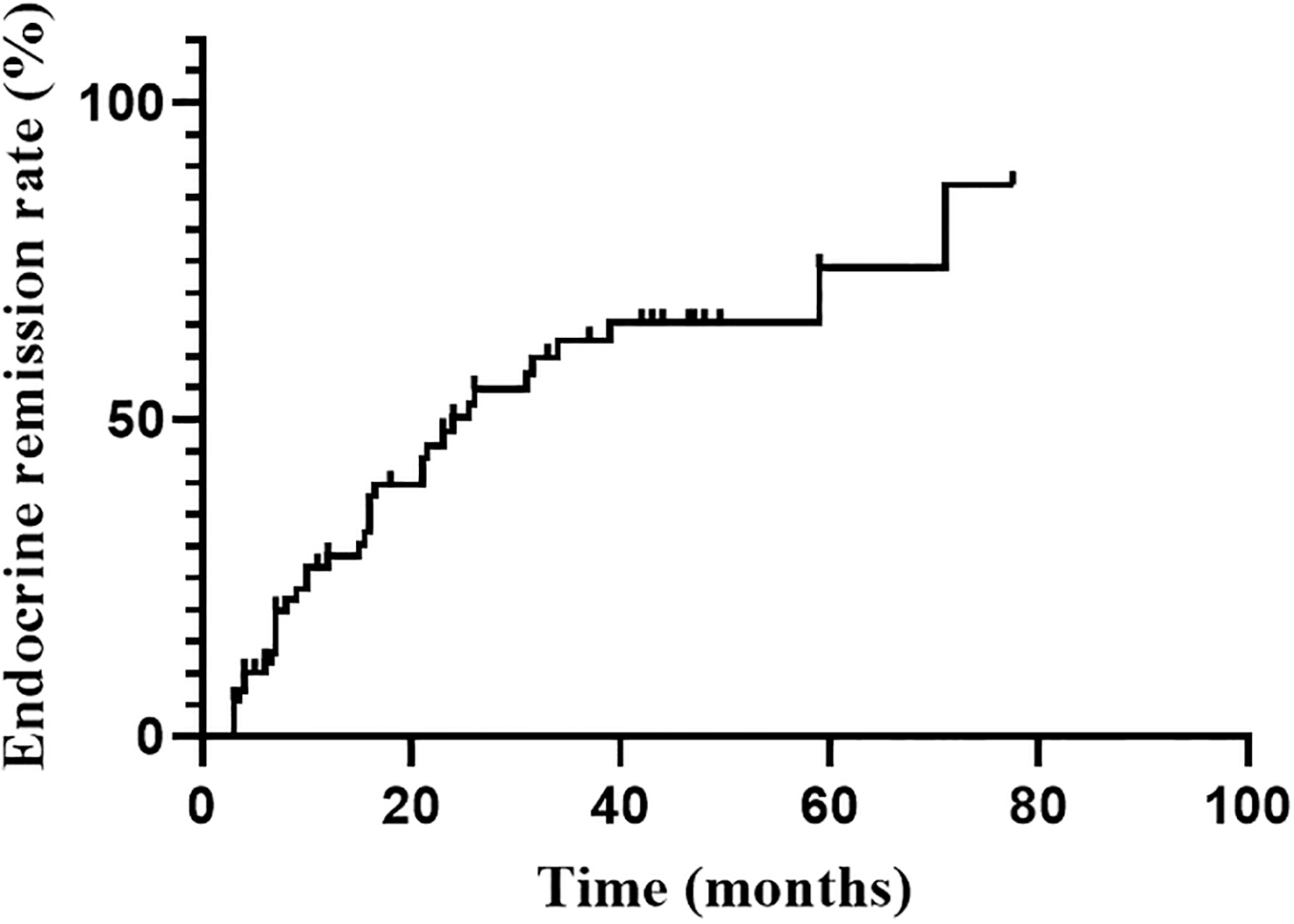
Endocrine remission rate during the follow-up after RT.

Endocrine remission was achieved in 37 of 70 patients during the follow-up. Six of them were evaluated by 1mg DST. The hormonal remission rate at 1, 2, 3 and 5 years were 28.5%, 50.2%, 62.5% and 74.0%, respectively, gradually increasing with follow-up time (**Figure 1**). The median time to remission was 24.0 months (95%CI: 14.0-34.0 months). Univariate analysis indicated that only higher Ki-67 index (P=0.044) was significant favorable factors for endocrine remission. There was no significant correlation between remission and age, sex, tumor size, the frequency of surgery, medication prior RT. The hormone levels (F, ACTH and 24hUFC prior RT) were divided into high and low groups by the median value, and were also not found to be associated with endocrine remission (**Table 2**). Since only Ki-67 was significant in the univariate analysis and all other parameters were far from significant, a multivariate analysis was no longer performed.

**Table 2:** Univariate predictors of endocrine remission.

	No. of pts.	MTR Months (95% CI)	P value
Age			
≤32 years	37	25.5 (5.7, 45.3)	0.659
>32 years	33	23.0 (19.2, 26.8)	
Sex			
Male	10	16.0 (0, 36.5)	0.241
Female	60	25.5 (14.7, 36.3)	
Residual tumors visible on MRI			
No	36	26.0 (14.2, 32.9)	0.490
Yes	34	21.0 (9.1, 32.2)	
Ki-67 index			
< 3%	32	39.0 (NA)	0.044
≥ 3%	20	21.0 (14.9, 27.1)	
ACTH			
Low	36	26.0 (14.9, 37.1)	0.803
High	34	21.5 (7.9, 35.1)	
F			
Low	36	21.5 (13.2, 29.8)	0.269
High	34	34.0 (15.0, 53.0)	
24hUFC			
Low	36	25.5 (14.4, 36.6)	0.827
High	34	23.0 (7.8, 38.3)	

MTR, median time to remission; HR, hazard ratio; RT, radiotherapy; NA, not available; ACTH, adrenocorticotropic hormone; F, serum cortisol; 24hUFC, 24-hour urinary free cortisol.

**Figure 2:**
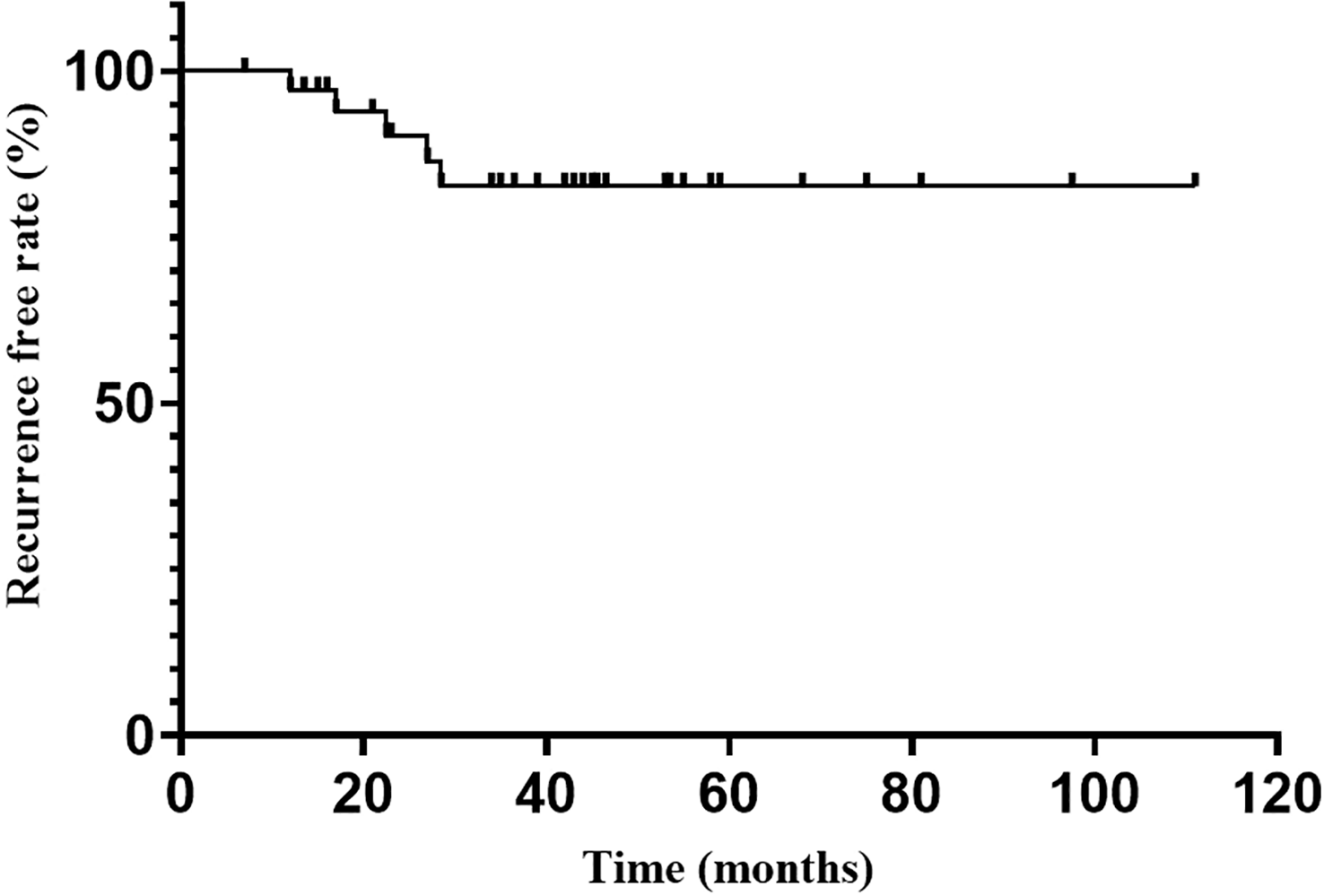
Recurrence free rate after endocrine emission.

Endocrine recurrence was found in 5 patients till the last follow-up, with an overall recurrence rate of 13.5% (5/37). The median time to recurrence after reaching endocrine remission was 22.5 months. The recurrence-free rate at 1, 2, 3 and 5 years after endocrine remission was 98.2%, 93.9%, 88.7% and 88.7%, respectively (**Figure 2**).

### Tumor control

A total of 51 patients had repeated MRI examinations before and after treatment. During the follow-up, 20 patients showed reduction and 30 patoents remained stable in tumor size, with a tumor control rate of 98%. Only 1 patient showed enlargement tumor 1 year after RT, with F, ACTH and 24hUFC increase continuously.

### Complications

At the last follow-up, 16 patients developed new onset hypopituitarism after RT. The overall incidence of RT-induced hypopituitarism was 22.9%. Hypothyroidism was the most common of hypopituitarism (8 patients), followed by HH (7 patients), adrenal insufficiency (4 patients) and GHD (3 patients). Only 1 patient (1.3%) with systemic lupus erythematosus (SLE) comorbidity complained of progressively worsening visual impairment during the follow up. No cerebrovascular event or radiation associated intracranial malignancy was found in our cohort.

## Discussion

### Efficacy and radiotherapy techniques

RT has been emerged as an effective second-line treatment for CD for many years. Although conventional fractionated RT has been used for a long experience in patients with CD, study on the modern precise radiotherapy, particularly IMRT, is rare and reports limited evidence on its long-term treatment outcome. IMRT can be implemented in many different techniques, such as fixed-field intensity-modulated radiotherapy (FF-IMRT), volumetric-modulated arc therapy (VMAT) and tomotherapy. Compared with conventional RT, IMRT allows a better target volume conformity while preserves adequate coverage to the target (14, 15). Our study reported that IMRT for CD has an endocrine remission rate of 74.0% at 5 years, with a median time to remission of 24.0 months (95%CI: 14.0-34.0 months). The endocrine remission rate at 5 years was comparable to those reported in previous series of FRT, with a median time to remission within the reported range (4.5-44 months) (9, 16–18) (**Table 3**). Compared with SRS in treating CD, the endocrine remission rate and median time to remission were also similar. Pivonello et al (19) summarized 36 studies of SRS for CD between 1986 to 2014, the mean endocrine remission rate was 60.8% and the median time to remission was 24.5 months. Tumor control rate was 98% in our cohort, only one patient showed enlargement tumor with elevating hormones. This local control rate was also comparable to that reported in a series of pituitary adenoma treated with FRT (93-100%) and SRS(92-96%) (9, 16–18, 20, 21). Indeed, despite the lack of controlled studies about SRS and FRT in treating CD, many reviews that summarize the biochemical control and tumor contral of both are similar (2, 6, 19).

**Table 3:** Literature review of FRT and SRS in patients with CD published in recent years.

Author Year	No. of patients	Type of RT	Dose	Remission criteria	Follow-up median (months)	Median/mean time to remission (months)	Remission rate at 5 years	Overall remission rate	Overall hypopituitarism rate	Recurrence rate after RT
Minniti 2007 (16)	40	Conventional RT	45-50Gy	1mg LDDST <50ng/ml or low 24h UFC level	108	24	78%	80%	75%	0%
Budyal 2014 (17)	20	CRT	45Gy	2mg LDDST<50nmol/L	37.5	20	65%	75%	40%	NA
*Pivonello R 2015 (19)	341	CRT	M: 43.5Gy R: 20-54Gy	NA	M: 81.9 R: 1-300	M: 17.4 R: 1-104	NA	M: 63.8% R: 19.6-100%	M: 39.3% R: 0-100%	M: 15.9% R: 0-62.5%
	850	SRS	M: 23.6Gy R: 14.7-45Gy (Margin Dose)		M:48.6 R: 2-264	M: 16 R: 1-166	NA	M: 60.8% R: 10-100%	M: 23.1% R: 0-66%	M: 12.3% R: 0-100%
Our study	70	IMRT	45-50Gy	LDDST<1.8ug/dl, 24hUFC normal	36.8	24	74%	NA	23.1%	13.5%

FRT, fractionated radiotherapy; SRS, stereotactic radiosurgery; LDDST, low dose dexamethasone suppressed test; CRT, conformal radiation therapy; M, mean; R, range; IMRT, intensity-modulated radiation therapy; NA, not available.

*Review study.

The overall endocrine recurrence rate in our study was 13.5%, with a median time to recurrence of 22.5 months. We, for the first time, reported the actuarial recurrence free rate at 1, 2, 3 and 5 years in CD patients treated with IMRT. The recurrence free rate at 3 and 5 years was 88.7% in our study. Outcomes were comparable to those reported in patients treated with conventional RT or SRS, with a mean recurrence rate and a median recurrence time of 15.9% (range, 0-62.5%) and 28.1 months, or 12.3% (range, 0-100%) and 33.5 months, according to a review conducted by Pivonello et al (19).

**Figure 3:**
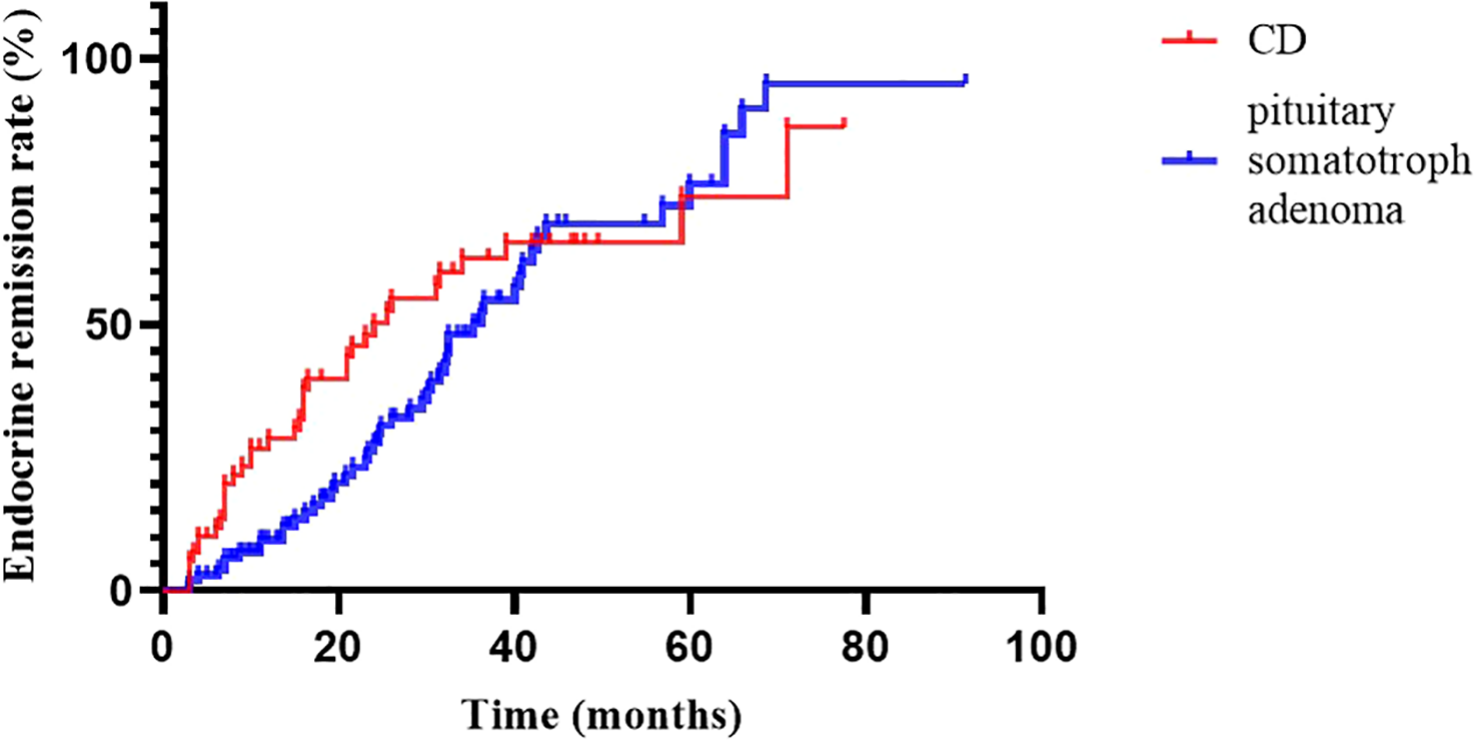
Endocrine remission rate of CD and pituitary somatotroph adenoma.

At 2020, we reported the outcomes of pituitary somatotroph adenomas treated with IMRT at our institution (20). Compared with pituitary somatotroph adenomas, CD has a similar 5-year remission rate (74.0% vs 74.3%) but a shorter median time to remission (24.0m vs 36.2m) (**Figure 3**). The tumor contral rates were similar, at 98% and 99%, respectively. The endocrine recurrence rate was significantly different, with CD being about one-fold higher than the pituitary somatotroph adenoma (13.8% vs 6.1%). This may be due to the majority of microadenomas in CD and that of macroadenomas in pituitary somatotroph adenomas.

### Predictors of endocrine remission

In the univariate analysis, we found that only Ki-67 index ≥ 3% was correlated with better endocrine remission (p=0.044). Cortisol levels before RT and tumor size were not predictors of endocrine remission. For surgery in treating CD, higher preoperative ACTH level was considered as unfavorable prognostic factor for endocrine remission in a few studies (22, 23). For radiotherapy, some previous studies also have reported a faster endocrine remission in patients with lower serum cortisol level. Minniti et al. reported that hormone level was normalized faster in patients with lower urinary and plasma cortisol level at the time of RT (16). Apaydin also reported that low postoperative cortisol and 1mg DST was a favorable factors for faster remission in patients treated with gamma knife surgery (GKS) and hypofractionated radiotherapy (HFRT), although no significant relationship was found between remission rate and plasma cortisol level prior RT in both studies (9, 16). Castinetti et al. found that initial 24hUFC was a predicative factor of endocrine remission in patients treated with GKS, which was not reported in our cohort treated with IMRT (24). However, the discrepancy between the results can be attributed to various factors, including selection bias of retrospective study, duration of follow-up, endocrine remission criteria and cut-off value.

Tumor size before RT was considered as a significant predictor for endocrine remission in some published series of patients treated with SRS. Jagannathan et al. reported a significant relationship between preoperative tumor volume and endocrine remission in patients with CD treated with GKS (25). However no significant correlation between tumor size and endocrine remission was found in series of patients treated with FRT (5, 9, 16, 17). But our study found no significant correlation between tumor size (visible or no-visible residual tumor on MRI) before RT and endocrine remission. The frequency of surgery before RT was also not found to be associated with endocrine remission in our study, which reached a similar conclusion with some previous studies (9, 17, 18, 26). Abu Dabrh et al. reported a higher remission rate in patients receiving TSS prior RT in their meta-analysis (5). Similar result was also reported in a review on the treatment outcome of GKS in patients with CD, that postoperative GKS was more effective than primary GK (19). However, analysis on this parameter was difficult in our cohort considering the low number of patients who received IMRT as the first-line treatment.

Reports on the effect of medical treatment on endocrine remission have been controversial. Some studies reported a negative effect of medical treatment at the time of SRS on endocrine remission in patients with CD. Castinetti et al. showed a significant higher rate of endocrine remission in patients who were not receiving ketoconazole at the time of GKS, compared to those who were (27). Sheehan et al. also found a significantly shorter time to remission in patients who discontinued ketoconazole at the time of GKS (28). However, no such correlation was found in patients treated with FRT (9, 17). Like previous studies on FRT, we also noted no significant relationship between preradiation use of medication and endocrine remission, but our statistical analysis may be hindered by the low proportion of patients undergoing medical treatment before RT. Moreover, the anticortisolic drugs used in previous studies were mainly ketoconazole or cabergoline, while most of our patient have received pasireotide, whose effect have not been well-studied yet. Further studies are necessary to understand the effect of somatostatin receptor ligands on the outcome of radiotherapy in patients with CD.

### Complications

Hypopituitarism is the most common complication secondary to radiotherapy, with the rate of new-onset hypopituitarism ranging widely in previous report. Pivonello et al. reviewed series of CD patients who were treated with conventional RT with a follow-up of at least 5 years (19). The reported mean and median rates of hypopituitarism were 50% and 48.3%, respectively (range, 0-100%). As regards FRT, the overall rate of new-onset hypopituitarism was 22.2-40% at a median follow-up ranging from 29-108 months, with both incidence and severity increasing with longer follow-up (9, 16–19). The incidence of hypopituitarism in our series was 22.9%, which was within the reported range of new onset hypopituitarism after FRT. Lower rate of hypopituitarism after SRS compared to conventional RT has been recognized in previous reviews (2, 6). Our study showed that new onset hypopituitarism was less prevalent after IMRT than after conventional RT. This can be attributed to a higher precision in contouring the target volume and OARs, allowing these modern radiotherapy techniques to provide a better protection to hypothalamus-pituitary axes. In previous studies, potential risk factors for new onset hypopituitarism included suprasellar extension, higher radiation dose to the tumor margin and lower isodose line prescribed (29, 30). Sensitivity of individual hormonal axes to RT varies in different series. In our study, central hypothyroidism was the most common individual axis deficiency, followed by HH, adrenal insufficiency and GHD. This sequence was similar to that reported by Sheehan et al., whose series included 64 CD patients treated with SRS, as well as some other series (29, 31). It is noted in some studies that GHD is the most vulnerable axes (19, 32, 33). Limited number of patients undergoing stimulation test may underestimate the prevalence of GHD in our study and some previous series, and longer follow-up is needed to generate a more accurate, time-dependent rate of new onset hypopituitarism.

In our study, only one patient complained of mild visual impairment, which was comparable to the rate ranging from 0-4.5% in previous series of FRT treating pituitary adenoma (9, 16–18, 26, 32, 34, 35). This patient had concomitant SLE and the associated microangiopathy may render the optic nerve intolerant to radiotherapy. Cranial nerve damage was acknowledged as an uncommon complication, with an estimated risk of vision deterioration below 1% if single radiation dose was no more than 2.0 Gy and total dose no more than 45-50 Gy (2, 36). The actuarial rate of optic neuropathy at 10 years was 0.8% in a series containing 385 patients with pituitary adenoma (37). No patient in our cohort developed cerebrovascular accident or secondary brain tumor. This finding was consistent with the low actuarial prevalence of these complications reported in other published series of FRT. Secondary brain tumor was extremely rare after SRS, with an overall incidence of 6.80 per patients-year, or a cumulative incidence of 0.00045% over 10 years in a multicenter cohort study containing 4905 patients treated with GKS (38). Ecemis et al. reviewed cohort studies of conventional RT in treating pituitary adenoma from 1990 to 2013 and found that 1.42% of patients developed secondary brain tumor, with a latency period of 19.6 years for meningioma, 11 years for glioma and 9 years for astrocytoma (39). As for cerebrovascular accident, Minniti et al. reported two patients (in a total of 40 patients) who had stroke 6 and 8 years after FRT (16). Data was still limited for FRT. Considering the low incidence and long latency period, large, controlled cohort study with long follow-up of FRT is still needed to accurately evaluate these complications.

### Limitations

Our study has several limitations. First, not all patients rigorously followed regular follow-up time points, making time-dependent statistical analysis less accurate. In addition, the excessively low number of cases with 1mg DST as the endocrine remission criterion may affect the accuracy of the remission rate.Moreover, a median follow-up time of about 3 years hampered evaluation on some late complications, including cerebrovascular events and secondary brain tumor.

In conclusion, our study revealed that IMRT was a highly effective second-line therapy with low side effect profile for CD patients, and it’s endocrine remission, tumor control and recurrence rates were comparable to previous reports on FRT and SRS.

## Data availability statement

The original contributions presented in the study are included in the article/supplementary material. Further inquiries can be directed to the corresponding author.

## Author contributions

1. Conceptualization: FZ and HZ 2. Data curation: XL and ZX. 3. Funding acquisition: FZ. 4. Investigation: XL and ZX 5. Methodology: WW 6. Resources: XL, SS and XH 7. Validation: LL and HZ. 8. Writing – original draft: ZX 9. Writing – review and editing: XL. All authors contributed to the article and approved the submitted version.
